# Molecular Mechanisms Underlying the Inhibition of Proliferation and Differentiation by Florfenicol in P19 Stem Cells: Transcriptome Analysis

**DOI:** 10.3389/fphar.2022.779664

**Published:** 2022-03-29

**Authors:** Dongfang Hu, Bin Zhang, Yu Suo, Zhiyue Li, Zhishuai Wan, Weihua Zhao, Lingli Chen, Zhihong Yin, Hongmei Ning, Yaming Ge, Weiguo Li

**Affiliations:** ^1^ Postdoctoral Research Station in Biological Sciences, Henan Normal University, Xinxiang, China; ^2^ College of Animal Science and Technology, Henan Institute of Science and Technology, Xinxiang, China; ^3^ Postdoctoral Research Base, Henan Institute of Science and Technology, Xinxiang, China

**Keywords:** florfenicol, embryonic toxicity, wnt pathway, hub genes, bioinformatics analysis

## Abstract

Florfenicol (FLO), which is widely used in veterinary clinics and aquaculture, can disrupt the protein synthesis of bacteria and mitochondria and, thus, lead to antibacterial and toxic effects in plants, insects, and mammals. FLO was found to repress chicken embryonic development and induce early embryonic death previously, but the underlying mechanism is not fully understood. Clarifying the mechanism of FLO-induced embryonic toxicity is important to the research and development of new drugs and the rational use of FLO to ensure human and animal health and ecological safety. In this study, the effects of FLO on pluripotency, proliferation, and differentiation were investigated in P19 stem cells (P19SCs). We also identified differentially expressed genes and performed bioinformatics analysis to obtain hub genes and conducted some functional analysis. FLO inhibited the proliferation and pluripotency of P19SCs and repressed the formation of embryoid bodies derived from P19SCs. A total of 2,396 DEGs were identified using RNA-Seq in FLO-treated P19SCs, and these genes were significantly enriched in biological processes, such as angiogenesis, embryonic organ development, and morphogenesis of organs. Kyoto encyclopedia of genes and genome-based pathway analysis also showed that five relevant pathways, especially the canonical Wnt pathway, were engaged in FLO-induced toxicity of pluripotent stem cells. We further analyzed modules and hub genes and found the involvement of ubiquitin-mediated proteolysis, DNA replication, and cell cycle machinery in regulating the pluripotency and proliferation of FLO-treated P19SCs. In summary, our data suggest that FLO disrupts the signaling transduction of pathways, especially the canonical Wnt pathway, and further inhibits the expression of target genes involved in regulating DNA replication, cell cycle, and pluripotency. This phenomenon leads to the inhibition of proliferation and differentiation in FLO-treated P19SCs. However, further experiments are required to validate our findings and elucidate the potential mechanisms underlying FLO-induced embryonic toxicity.

## 1 Introduction

Several kinds of antimicrobials have been applied in human medicine and agriculture to prevent or treat animal and plant bacterial infections over the past decades. Moreover, antimicrobials can promote the growth of mammals, birds, and fish when used as feed additives (Schar et al., 2020). The successful use of antimicrobials has significantly increased the production and use of these compounds (Tiseo et al., 2020; Tian et al., 2021). Previous studies point out that 73% of all antimicrobials sold worldwide are used in food-producing animals (Van Boeckel et al., 2017), which is related to drug-resistant infections in animals and humans (Jibril et al., 2021; Tian et al., 2021). In the production and various applications of a mass of antimicrobials, they are released mainly through wastewater, biosolids, and animal feces and can affect ecosystems and biogeochemical processes (Zhou et al., 2021).

The bacterial ribosome becomes an important target of varieties of clinically useful antimicrobials (Lade and Kim 2021). Mitochondria and chloroplasts have bacterial-type ribosomes because they are evolved from endosymbiotic α-proteobacteria and cyanobacteria-like prokaryotes, respectively (Ferrari et al., 2021); this condition makes these organelles vulnerable to ribosome-targeted antimicrobials (Wang et al., 2015). Increasing numbers of studies show that some intensively used antimicrobials disturb mitochondrial proteostasis and physiology in animals ranging from worms, fruit flies, and mice to human cell lines (Wang et al., 2015), and they also impact plant chloroplasts, inducing toxic effects on terrestrial and aquatic plants (Carvalho et al., 2014; Minden et al., 2018).

Florfenicol (FLO), which is a derivative of chloramphenicol and thiamphenicol, is widely used in veterinary clinics and aquaculture against bacterial infections and to promote animal growth. The drug can reversibly bind to the large subunit of the bacterial ribosome and mitoribosome and inhibit peptidyl transferase in prokaryotic organisms and mitochondria, which leads to antibacterial effects and mitochondrial protein synthesis inhibition (Hu et al., 2017). FLO is widely used to prevent and treat bacterial diseases of livestock, poultry, and aquatic animals (Morrow et al., 2020); it can also reduce the generation of inflammatory factors and inhibit inflammatory response; therefore, it can be applied in septic shock and respiratory inflammation (Er and Dik 2014).

Overuse or misuse of FLO is reported to promote the generation of antibiotic-resistant bacteria (Abdelfattah et al., 2021; Choi et al., 2021), which leads to antibiotic contamination or residue in animal products (Bortolotte et al., 2021), agricultural soils (Ma et al., 2021), drinking water sources (Feng et al., 2020), and increased environmental risk. More recent studies show that FLO, which is an antimicrobial especially used in veterinary clinics, can be frequently detected in the urine of pregnant women and children, which leads to a disturbance of the microbiome in pregnant women and childhood obesity (Wang et al., 2017; Zhou et al., 2021). The side effects of FLO are emerging and include but are not limited to immunotoxicity, reproductive toxicity, and embryonic toxicity (Guan et al., 2011; Al-Shahrani and Naidoo 2015; Hu et al., 2020). The embryonic toxicity of FLO is confirmed by veterinary clinical practice (Al-Shahrani and Naidoo 2015) and animal experimental research (Hu et al., 2020). Whether continuous exposure of pregnant women to low-dose FLO causes other toxic and side effects other than microbiome disturbance and childhood obesity is unclear.

In our previous study, we found that FLO repressed chicken embryonic development and induced early embryonic death (Hu et al., 2020) although the mechanisms underlying FLO-induced embryonic toxicity are not fully understood. P19 stem cells (P19SCs), which are the malignant counterparts of embryonic stem cells (ESCs), are pluripotent embryonal carcinoma cells and considered a good model to study stem cell differentiation; they can differentiate into the three germ layers (Vega-Naredo et al., 2014). Therefore, P19SCs are a useful system for the molecular and cellular studies associated with developmental toxicity (Seeley and Faustman 1998). To understand the molecular mechanisms involved in chemical-induced toxicity, high-throughput sequencing technologies, including RNA sequencing analysis (RNA-Seq), are indispensable tools for analyzing differentially expressed genes (DEGs) at the transcriptome level (Stark et al., 2019).

In this study, we selected P19SCs as a model system to investigate the toxic effects of FLO and the underlying mechanisms at the transcriptional level. The effects of FLO on the proliferation, pluripotency and global transcription, and cardiac differentiation are investigated in P19SCs. We also identified DEGs, performed bioinformatics analysis to obtain hub genes, and conducted some functional analysis. Our research provides a basis for understanding mechanisms underlying FLO-induced embryonic toxicity and helps guide the rational use of FLO to ensure human and animal health and ecological safety.

## 2 Materials and Methods

### 2.1 Chemicals

Minimum essential medium α (αMEM) and penicillin–streptomycin solution (100×) were from Gibco (Life Technologies, CA). Bovine calf serum (CS) and fetal calf serum (FBS) were from Biological Industries (Kibbutz BeitHaemek, Israel). FLO was from Aladdin (Shanghai, China). DMSO was from Sigma (St. Louis, MO, United States). Sox2 (66411-1-Ig), Oct4 (11263-1-AP), β-catenin (66379-1-Ig), and β-actin (66009-1-Ig) were from Proteintech (Wuhan, China).

### 2.2 Cell Culture

P19SCs (cat.no. CL-0179) were obtained from Procell Life Science & Technology Co., Ltd. (Wuhan, China), and they were maintained in αMEM medium (7.5% CS, 2.5% FBS, 1 IU/ml penicillin, and 1 μg/ml streptomycin). Cells were cultured at 37°C with 5% CO_2_.

### 2.3 Florfenicol Treatment

FLO solution was prepared in accordance with our previous protocol (Hu et al., 2017). Previous studies find that FLO can cause early chicken embryonic death and growth retardation, and the embryotoxic concentration of FLO in eggs ranges from 3.51 μg/g to 5.03 μg/g when laying hens were given FLO at 90 mg/kg daily for 5 days (Al-Shahrani and Naidoo 2015; Hu et al., 2020). Moreover, studies on the pharmacokinetics of FLO show that the peak plasma concentrations of FLO range from 5.21 μg/ml to 10.71 μg/ml after a single oral administration in rabbits and dogs (Park et al., 2007; 2008). Thus, the FLO levels used in this study (1.56–25 μg/ml) are similar to those that a developing embryo or cells might encounter.

### 2.4 Differentiation of P19SCs

Cardiac mesodermal differentiation was induced according to a reported protocol (Hoque et al., 2018). Briefly, P19SCs were trypsinized and suspended in differentiation medium (growth medium containing 1% DMSO) with FLO at a density of 500 cells/20 μL drop. The drops of cell suspension were placed on the upturned inner surface of the lid of a Petri dish and cultured for 2 days (day 2). Then, each drop was transferred to a 96-well ultralow attachment plate containing fresh differentiation medium with FLO. After 3 days (day 5), the embryoid bodies (EBs) were transferred to a gelatin-coated plate containing fresh differentiation medium with FLO.

### 2.5 Cell Proliferation and Death Analysis

P19SCs were plated in 96-well plates and treated with multiple doses of FLO for 24 or 48 h to determine the effects of FLO on stem cell proliferation and survival. The medium was removed, and 100 μL PBS containing 10 μL CCK-8 solution (TransGen Biotech, China) was added into each well and maintained at 37°C. After 2 h, the absorbance was determined at 450 nm for calculating the percentage of cell viability. Lactate dehydrogenase (LDH) released into the culture medium as a marker of cell death was measured using the LDH Activity Assay Kit (Nanjing Jiancheng, Nanjing, China) according to the instructions of the manufacturer.

### 2.6 RNA Isolation and qPCR

Total RNA were extracted using Trizol reagent and reverse transcribed using RT Easy™ II (Foregene, Chengdu, China) according to the protocol of the manufacturer. Real-time qPCR was performed using the SYBR Green PCR Kit (Qiagen, Hilden, Germany), and specific primers were used (Supplementary Table S1). Samples were run in triplicate, and gene expression levels were analyzed and normalized with GAPDH using the comparative threshold method (2^‐△△Ct^).

### 2.7 Western Blot

The treated cells were washed with cold PBS and lysed using RIPA reagent (supplemented with protease inhibitors) (Thermo Scientific, Waltham, MA). Equal amounts of protein from each sample were loaded and electrophoresed. The proteins were transferred to PVDF membranes and incubated with specific antibodies (β-actin (1:5,000), Sox2 (1:1,000), Oct4 (1:1,000), and β-catenin (1:2,000)). Then, immune complexes were detected with secondary antibody (Cwbiosciences, Beijing, China) and visualized by enhanced chemiluminescence (Pierce, Appleton, Wisconin). Average densitometry values were determined using Image J software and were normalized by β-actin as a loading control.

### 2.8 Immunostaining

P19SCs were exposed to 0, 1.56, 6.25, and 25 μg/ml FLO to understand the influences of FLO on the formation of EBs and the expression of β-catenin. At the seventh day of differentiation, the differentiated aggregates in the 96-well plates were fixed using 4% paraformaldehyde solution, permeabilized with 0.2% Triton X-100, and washed using PBS. After aggregates were blocked with 1% BSA, they were incubated at 4°C overnight with β-catenin antibodies (1:500 dilution). After aggregates were washed, they were incubated with FITC-conjugated secondary antibodies at 37°C for 1 h. DAPI (Invitrogen) was used to stain the nuclei. Images were taken using a confocal microscope. The relative size of EBs, mean gray value, and integrated density of β-catenin were quantified using Image J software.

### 2.9 Library Construction and Sequencing

Total RNA was extracted, and the RNA integrity was measured using the Agilent 2100 bioanalyzer system. Libraries of amplified RNA for each of the six samples were prepared in accordance with the Illumina protocol. The clustering of the index-coded samples was performed using TruSeq PE Cluster Kit v3-cBot-HS (Illumia). After cluster generation, the library preparations were sequenced and 150 bp paired-end reads were generated (Novogene, Beijing, China).

### 2.10 Identifying DEGs From RNA-Seq Data

Reads containing adapter or ploy-N or that showed low quality were removed from the raw data to generate clean reads for further analysis. Paired-end clean reads were aligned to the reference genome using Hisat2 (v2.0.5). The mapped reads of each sample were assembled by StringTie (v1.3.3b). The numbers of reads mapped to each gene were counted using FeatureCounts (v1.5.0). Based on the length of the gene and reads count mapped to this gene, fragments per kilobase of exon model per million mapped fragments (FPKM) of each gene was calculated. The DESeq2 R package (1.20.0) was used to analyze DEGs. Genes with an adjusted *p* value (padj) <0.05 and |log2 (foldchange)| > 0.585 were assigned as DEGs. The RNA-sequencing data has been deposited in the Genome Sequence Archive in the National Genomics Data Center, China National Center for Bioinformation/Beijing Institute of Genomics, Chinese Academy of Sciences, under accession number CRA005030 (https://ngdc.cncb.ac.cn/gsa).

### 2.11 Functional Analysis of DEGs

The clusterProfiler R package was used to perform Gene Ontology (GO) enrichment analysis of DEGs. KEGG (http://www.genome.jp/kegg/)-based pathway analysis was performed using the clusterProfiler R package and DAVID database (v6.8, https://david.ncifcrf.gov/). The GO terms and KEGG pathways with padj less than 0.05 were considered significantly enriched by DEGs.

### 2.12 Protein–Protein Interaction Network Construction and Module Analysis

DEG-encoded proteins and the protein–protein interaction (PPI) were generated using the online STRING database (v11.0, https://www.string-db.org/). An interaction with a combined score ≥0.4 was considered statistically significant. Cytoscape (v3.8.2) with the Molecular Complex Detection (MCODE) app (v2.0.0) was used to cluster a given network based on the topology to find densely connected regions (Bader and Hogue 2003) with a degree cutoff = 2, node score cutoff = 0.2, k-score = 2, and max depth = 100. The genes involved in these modules were further used to perform GO annotation and KEGG pathways analysis using the DAVID database.

### 2.13 Hub Gene Selection and Functional Analysis

The hub genes of the upregulated or downregulated DEGs were identified with the cytoHubba app (v0.1) from Cytoscape using the degree method. The KEGG pathways enriched by hub genes were performed using the DAVID database (*p* value <0.05).

### 2.14 Statistical Analysis

Data are shown as the mean ± SD, and they were analyzed using one-way ANOVA followed by the LSD multiple comparison test (SPSS, Chicago, IL). A *p* value less than 0.05 was considered statistically significant.

## 3 Results

### 3.1 Florfenicol Inhibits the Proliferation and Pluripotency of P19SCs

**FIGURE 1:**
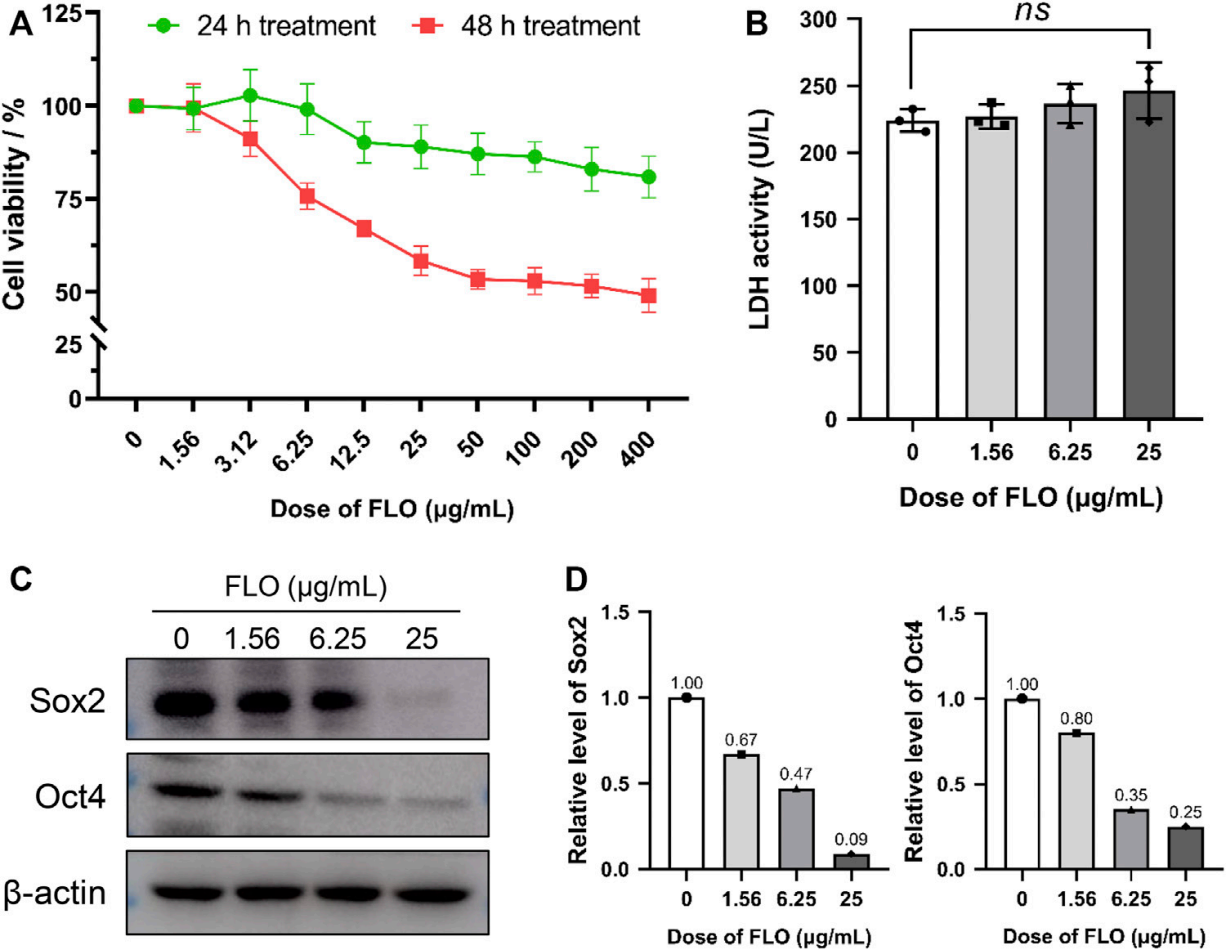
FLO inhibits the proliferation and pluripotency of P19SCs. **(A)** P19SC viability in response to FLO-treatment as detected by CCK-8 method. The results are expressed as the average cell viabilities from three independent experiments. **(B)** Effect of different doses of FLO on LDH release of P19SCs when exposed for 48 h; *ns*, not significant. **(C)** Immunoblot analysis of multiple doses of FLO on protein levels of pluripotent markers (Sox2 and Oct4) in P19SCs treated for 48 h.

We previously found that FLO could inhibit the development of chick embryos and induce early embryonic death. To understand the influences of FLO on stem cell survival and proliferation during embryonic development, P19SCs were treated with FLO for 24 or 48 h. As shown in Figure 1A, FLO inhibited cell proliferation in a dose- and time-dependent manner. The supernatant collected from FLO treated and untreated cells were subjected to LDH detection to further determine the effect of FLO on cell death. The results show that FLO did not significantly induce LDH release and cell death at the dose of 25 μg/ml (*p* > 0.05) (Figure 1B). Our results suggest that FLO could inhibit the self-renewal of P19SCs without causing obvious cell death.

Transcription factors Sox2 and Oct4 are essential in maintaining the pluripotency of ESCs and conferring stemness in stem cells. We detected the expression level of these pluripotent markers to clarify the effect of FLO on the pluripotency and stemness of P19SCs. The results show that FLO significantly inhibited the protein expression of Sox2 and Oct4 (Figure 1C), which indicated repressed pluripotency in FLO-treated P19SCs.

### 3.2 Illumina Hiseq mRNA Sequencing

The cells were treated with 10 μg/ml FLO for 48 h, and Illumina Hiseq mRNA sequencing was performed to explore the mechanism underlying FLO-induced stem cell toxicity and assess the overall gene changes in P19SCs post FLO treatment. In summary, 270 million raw reads were generated from the six libraries. A transcriptome for P19SCs of 39.36 Gb was assembled using 262 million clean reads having read lengths of 150 bp from FLO treated and untreated cells. The sequence reads with the GC (%) of 51.14–52.52 and Phred quality score (Q30) of 94.08%–94.56% indicated that the reads were of high quality. A total of 91.28%–92.70% of the clean reads were aligned against the *Mus musculus* (GRCm38. p6) reference genome, among which, 94.52%–96.47% were mapped to exonic regions, 1.48%–3.09% to intronic regions, and 2.05%–2.75% to intergenic regions (Supplementary Table S2).

### 3.3 Differential Expression Analysis

**FIGURE 2:**
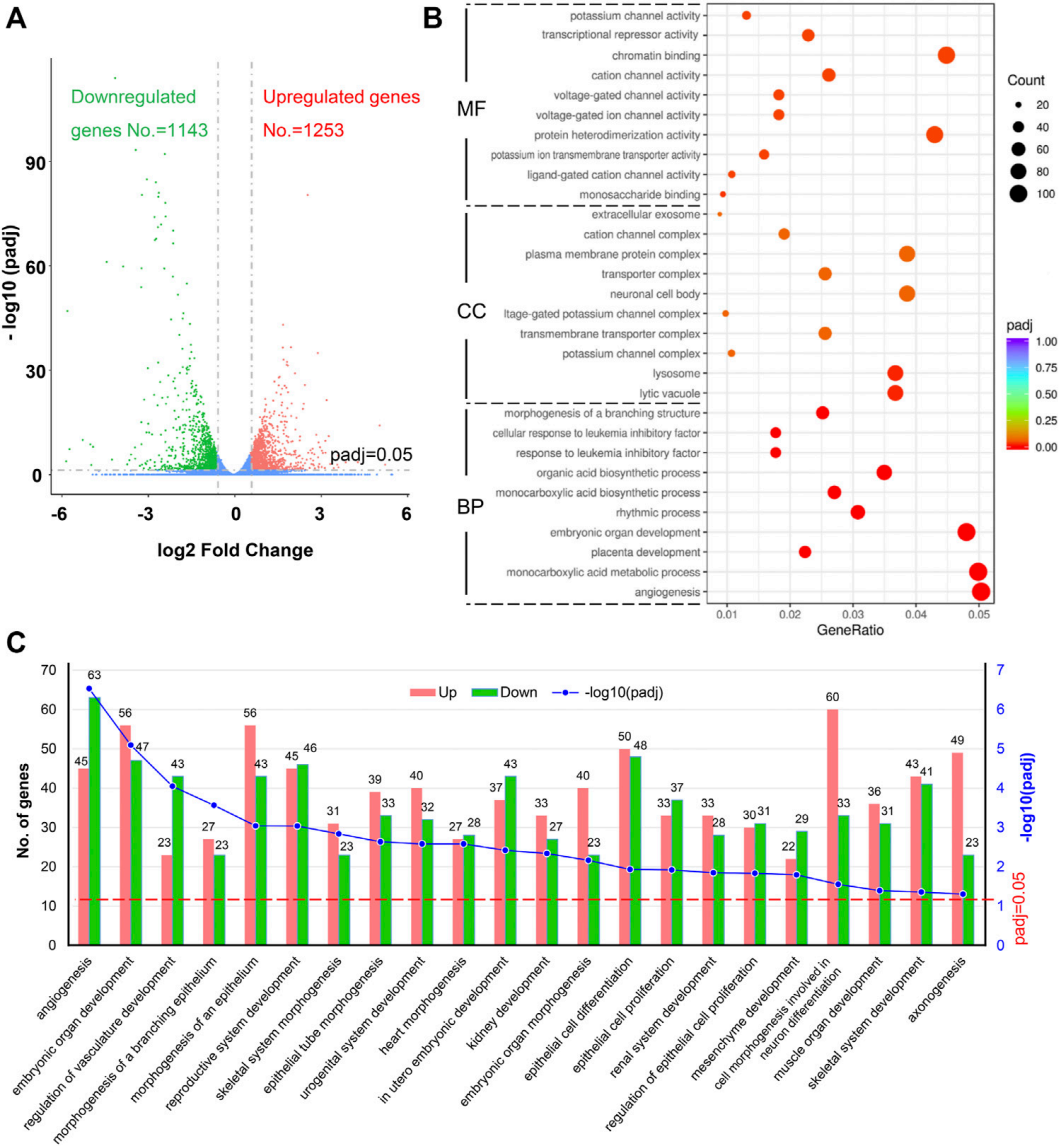
Identification of DEGs and GO enrichment analysis. **(A)** Volcano plot of DEGs. Upregulated genes (padj <0.05 and fold change >1.5) are shown in red, and downregulated genes are shown in green (padj <0.05 and fold change <0.66). padj, adjusted *p* value. **(B)** Top 10 enriched GO terms of BP, CC, and MF sorted according to padj value. GeneRatio indicates the ratio of enriched DEGs over total DEGs. The size and color of the dot indicate the count of DEGs enriched in the GO term and the significance, respectively. **(C)** Significantly enriched BP terms related to embryonic development, which were enriched by more than 50 DEGs, are shown. The numbers of upregulated and downregulated DEGs related to each BP term are indicated.

The DEGs were identified among the samples. The mean value of the normalized read count values in each group was used to calculate the mRNA expression ratio between FLO treated and untreated cells. A ratio with a fold change (FC) > 1.50 or <0.66 and padj <0.05 in the FLO group compared with that of the control group was considered a DEG. We identified 2,396 DEGs, of which 1,253 and 1,143 were upregulated and downregulated, respectively, in the FLO group (Figure 2A). Among the unregulated DEGs, 85 genes were above 4 FC, 413 genes were between 2 and 4 FC, and 755 genes were between 1.5 and 2 FC. For the downregulated genes, 120 genes were below 0.25 FC, 351 genes were between 0.25 and 0.5 FC, and 672 genes were between 0.5 and 0.66 FC (Supplementary Figure S1).

### 3.4 GO Functional Analysis of DEGs

The ClusterProfile tool was used to analyze the dysregulated genes for revealing the biological processes (BP), cell components (CC), and molecular functions (MF) altered in FLO-treated P19SCs to achieve global insights into the 2,396 DEGs. A total of 5,390 BPs were enriched in this study, and 204 were statistically significant with padj <0.05 (Supplementary Figure S2). The top 10 significantly enriched terms sorted according to padj value are shown in Figure 2B. We further identified 22 significantly enriched BP terms that were enriched by more than 50 DEGs and related to embryonic development, including angiogenesis, embryonic organ development, regulation of vasculature development, and morphogenesis of organs (Figure 2C). For CC ontology, 630 components were enriched, but only lytic vacuole and lysosome were statically significant (padj <0.05). With regard to MF ontology, 964 MF terms were enriched and 17 were statically significant (padj <0.05) (Supplementary Figure S2). The top 10 significantly enriched MF terms are shown in Figure 2B. The key functions of changed genes were associated with monosaccharide binding, ligand-gated cation channel activity, potassium ion transmembrane transporter activity, protein heterodimerization activity, voltage-gated ion channel activity, and others.

### 3.5 KEGG Pathway Analysis of DEGs

**FIGURE 3:**
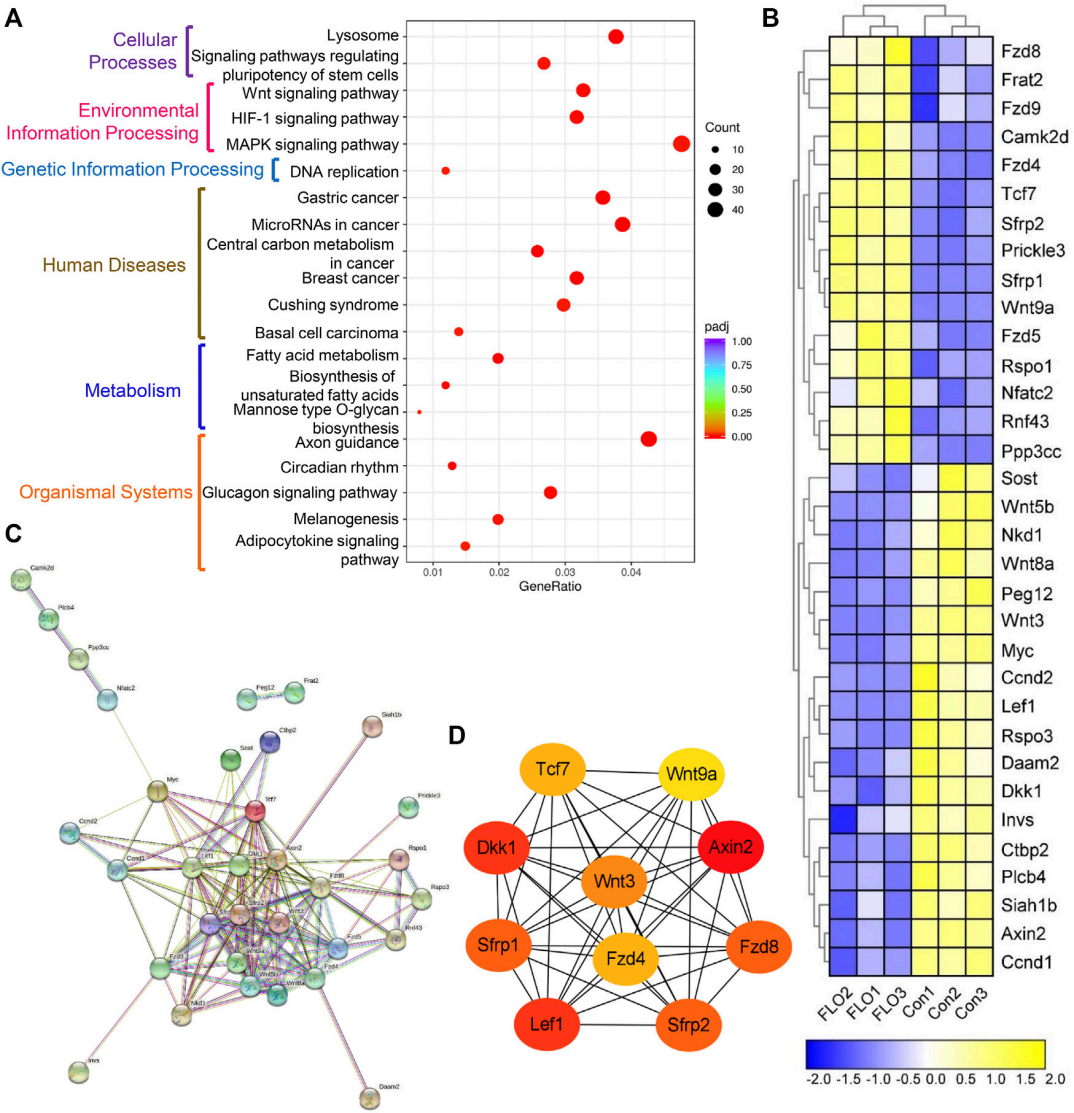
KEGG pathway enrichment analysis of the integrated DEGs. **(A)** The top 20 of the 45 significantly enriched KEGG pathways clustered into six subcategories. GeneRatio indicates the ratio of enriched DEGs over total DEGs. The size and color of the dot indicate the count of DEGs enriched in the KEGG pathway and the significance, respectively. **(B)** Heat map of the DEGs enriched in the Wnt pathway. Expression values of the six libraries are presented as the normalized FPKM values. Yellow colors indicate upregulated DEGs. Blue colors indicate downregulated DEGs. **(C)** PPI network analysis of the 33 DEGs based on the String database. **(D)** Top 10 hub genes in the network ranked by the degree method using cytoHubba.

KEGG-based pathway analysis was performed to collect information regarding protein functions in metabolic processes to achieve the specific biological events of DEGs. A total of 45 significantly enriched KEGG pathways were obtained, and the top 20 of them were clustered into six subcategories as shown in Figure 3A. Among these pathways, we focused on the Wnt signaling pathway, which is an evolutionarily conserved pathway involved in the proliferation and maintenance of stem cells and embryogenesis. A total of 33 DEGs were enriched in the Wnt pathway, including 15 upregulated and 18 downregulated genes (Figure 3B). Three key genes in the Wnt pathway (Wnt3, Wnt8a, and Myc) and three other genes were chosen to verify the RNA-Seq results by qRT-PCR methods (Supplementary Figure S3). The qRT-PCR results show that the expression patterns of the six genes were consistent with the results of RNA-Seq, which indicates that the RNA-Seq results are reliable.

**FIGURE 4:**
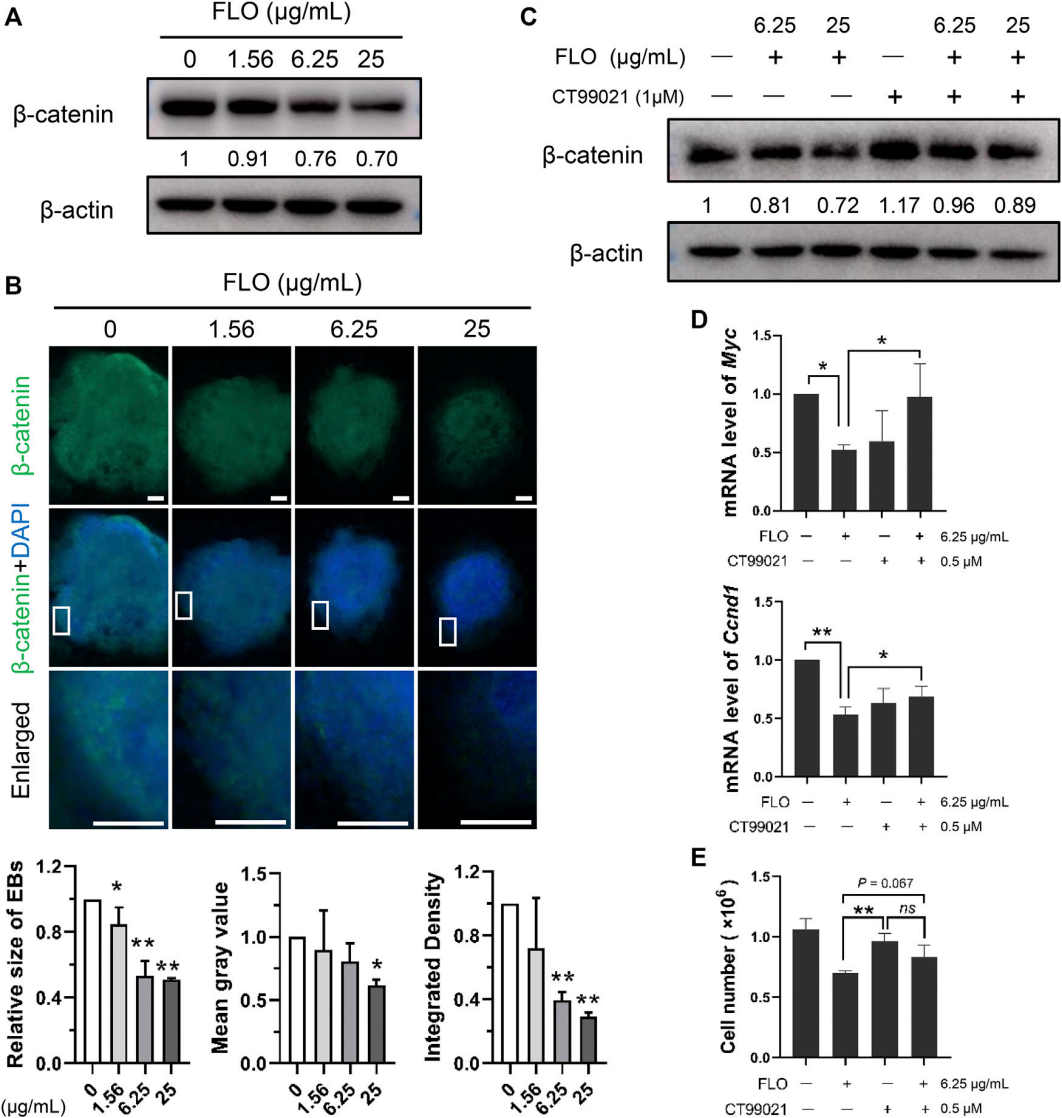
The canonical Wnt pathway is engaged in mediating FLO-induced toxic effects. **(A)** Immunoblot analysis of FLO on protein levels of β-catenin in P19SCs treated for 48 h β-actin served as a loading control. Densitometry was performed for quantification and the ratios of β-catenin to β-actin are presented at the bottom of the blots. **(B)** Immunostaining of the differentiated EBs (day 7) using FITC-conjugated β-catenin antibodies. Representative images are shown to demonstrate the influences of FLO on the formation of EBs and the expression of β-catenin. DAPI was used to stain cell nucleus. Bar = 200 μm. The relative size of EBs, mean gray value, and integrated density of β-catenin were quantified using Image J software. **(C)** Immunoblot analysis of CT99021 (activator of the canonical Wnt pathway) on β-catenin level in FLO-treated P19SCs. **(D)** Effects of CT99021 on mRNA expression levels of Myc and Ccnd1. **(E)** The influence of CT99021 on the proliferation of FLO-treated P19SCs. 5 × 10^4^ cells were cultured in each well of 12-well plates for 12 h. Then cells were treated with FLO (6.25 μg/ml) and CT99021 (0.5 μM) for another 48 h. The results are expressed as the average cell numbers from three independent experiments. *p < 0.05, **p < 0.01, as compared with the control group. *ns*, not significant.

We did a PPI network analysis of the 33 DEGs based on the String database to further understand the function of the Wnt pathway. Ultimately, the PPI network included 33 nodes and 149 edges (Figure 3C), which exhibited significantly more interactions than expected with a PPI enrichment *p* value <1.0E-16. We then visualized the PPI network using Cytoscape and selected hub genes using cytoHubba. The top 10 genes in the network ranked by the degree method were Axin2, Dkk1, Lef1, Sfrp2, Sfrp1, Fzd8, Wnt3, Fzd4, Tcf7, and Wnt9a, which were key molecules of the canonical Wnt pathway (Figure 3D). We then analyzed the expression profile of β-catenin, which is the most critical and core signal transduction factor of the canonical Wnt pathway, in FLO-treated P19SCs and the derived EBs. The results show that FLO reduced the expression of β-catenin in a dose-dependent manner (Figure 4A). In addition, FLO significantly inhibited the formation of EBs derived from P19SCs, and a twofold decrease in EBs size post 25 μg/ml FLO treatment was achieved (Figure 4B). We also found a dramatic decrease in the level of β-catenin post FLO treatment (Figure 4B). Activation of the canonical Wnt pathway using CT99021 significantly increased the protein level of β-catenin (Figure 4C), promoted the mRNA expression levels of Myc and Ccnd1 (*p* < 0.05, Figure 4D), and partially reversed the inhibitory effect of FLO on P19SCs proliferation (*p* = 0.067, Figure 4E). Collectively, these results indicate that the canonical Wnt pathway plays a key role in the proliferation and differentiation toxicity of P19SCs induced by FLO, but the specific mechanisms need further study.

### 3.6 PPI Network Construction and Module Analysis

**FIGURE 5:**
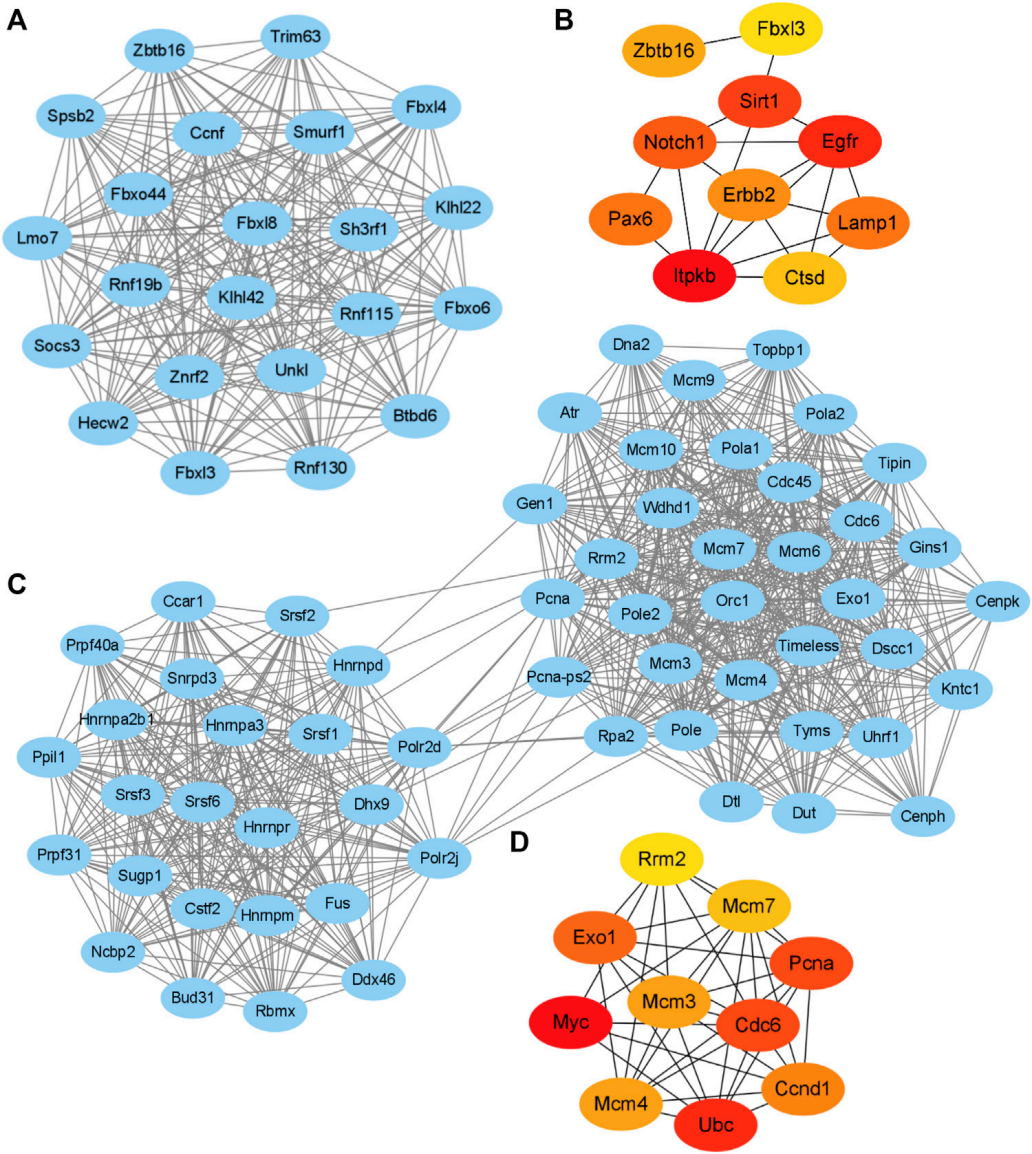
Key modules and hub gene analysis of the DEGs. The PPI networks were constructed and the most significant modules of the upregulated DEGs **(A)** and downregulated DEGs **(C)** were obtained using MCODE app from Cytoscape. The top 10 hub genes of the upregulated **(B)** and downregulated DEGs **(D)** according to node degree were achieved using cytoHubba app from Cytoscape, respectively. The darker color of the dot indicates higher score and higher importance of the gene.

The String database and Cytoscape software were used to merge the 1,237 known upregulated genes and 1,124 known downregulated genes, respectively, for identifying the significant modules. The PPI networks of the upregulated and downregulated DEGs were constructed (Supplementary Figures S4, S5), and the most significant modules were obtained using the MCODE app from Cytoscape (Figures 5A,C). The following GO and KEGG pathways were analyzed using genes involved in these modules based on the DAVID database. Enrichment analyses for the upregulated DEGs showed that changes in BP of the significant module genes were mainly enriched in protein ubiquitination, proteasome-mediated ubiquitin-dependent protein catabolic processes, and protein ubiquitination involved in ubiquitin-dependent protein catabolic processes. Changes in CC of the significant module genes were significantly enriched in the SCF ubiquitin ligase complex, cytoplasm, and cytosol. Changes in MF of the significant module genes were significantly enriched in ubiquitin-protein transferase, ligase, and ubiquitin protein ligase activities. KEGG pathway analysis reveals that the significant module genes are enriched in ubiquitin-mediated proteolysis (*p* = 8.90E-02) (Table 1).

**TABLE 1:** GO and KEGG pathway enrichment analysis of the significant module of upregulated DEGs.

Category	Term^a^	Count	%	*p* Value
BP	protein ubiquitination	9	40.9	5.00E-10
	proteasome-mediated ubiquitin-dependent protein catabolic process	6	27.3	2.00E-07
	protein ubiquitination involved in ubiquitin-dependent protein catabolic process	5	22.7	6.40E-06
	SCF-dependent proteasomal ubiquitin-dependent protein catabolic process	3	13.6	2.50E-04
CC	SCF ubiquitin ligase complex	5	22.7	3.70E-07
	cytoplasm	18	81.8	3.40E-06
	cytosol	7	31.8	7.00E-03
MF	ubiquitin-protein transferase activity	14	63.6	6.50E-19
	Ligase activity	9	40.9	2.00E-09
	ubiquitin protein ligase activity	6	27.3	1.90E-06
	zinc ion binding	8	36.4	8.60E-05
KEGG pathway	Ubiquitin mediated proteolysis	2	9.1	8.90E-02

a The top four terms were listed on the basis of p value if over four terms were enriched in the category.

Enrichment analyses for the downregulated DEGs show that changes in BP of the significant module genes were mainly enriched in DNA replication, DNA replication initiation, and RNA splicing. Changes in CC of the significant module genes were mainly enriched in the nucleus, nucleoplasm, and spliceosomal complex. Changes in MF of the significant module genes were significantly enriched in nucleotide, DNA, and RNA binding. KEGG pathway analysis reveals that the significant module genes were significantly enriched in DNA replication, spliceosome, pyrimidine metabolism, and cell cycle (Table 2).

**TABLE 2:** GO and KEGG pathway enrichment analysis of the significant module of downregulated DEGs.

Category	Term^a^	Count	%	*p* Value
BP	DNA replication	21	36.2	8.70E-30
	DNA replication initiation	10	17.2	2.00E-17
	RNA splicing	15	25.9	1.80E-14
	mRNA processing	16	27.6	4.70E-14
CC	nucleus	56	96.6	3.40E-26
	nucleoplasm	34	58.6	3.00E-19
	spliceosomal complex	11	19	5.90E-12
	catalytic step 2 spliceosome	8	13.8	1.10E-08
MF	nucleotide binding	28	48.3	4.10E-12
	DNA binding	27	46.6	1.00E-11
	RNA binding	18	31	1.80E-10
	poly(A) RNA binding	20	34.5	7.40E-10
KEGG pathway	DNA replication	11	19	1.60E-16
	Spliceosome	13	22.4	2.60E-13
	Pyrimidine metabolism	9	15.5	1.10E-08
	Cell cycle	9	15.5	5.90E-08

a The top four terms were listed on the basis of p value if over four terms were enriched in the category.

### 3.7 Hub Gene Selection and KEGG Functional Analysis

The hub genes of the upregulated or downregulated DEGs were identified with the cytoHubba app from Cytoscape. The 10 most significant upregulated genes according to node degree were Itpkb, Egfr, Sirt1, Notch1, Lamp1, Pax6, Erbb2, Zbtb16, Ctsd, and Fbxl3 (Figure 5B and Supplementary Table S3). The 10 most significant downregulated genes according to node degree were Myc, Ubc, Cdc6, Pcna, Exo1, Ccnd1, Mcm4, Mcm3, Mcm7, and Rrm2 (Figure 5D and Supplementary Table S3). The KEGG pathways of these hub genes were reanalyzed via DAVID to better understand their functions. Among the top significantly enriched pathways, cell cycle was enriched by seven downregulated hub genes, including Cdc6, Ccnd1, Mcm3, Mcm4, Mcm7, Myc, and Pcna. Mcm3, Mcm4, Mcm7, Myc, and Pcna were also enriched in DNA replication (Table 3). These results suggest that abnormal regulation of DNA replication and cell cycle may play important roles in FLO-induced P19SC toxicity.

**TABLE 3:** KEGG enrichment analysis of the hub genes.

KEGG pathway	Count	%	*p* Value	Hub genes
Cell cycle	7	35	3.60E-07	Cdc6, Ccnd1, Mcm3, Mcm4, Mcm7, Myc, Pcna
DNA replication	4	20	8.00E-05	Mcm3, Mcm4, Mcm7, Myc, Pcna
Thyroid hormone signaling pathway	3	15	3.20E-02	Ccnd1, Myc, Notch1
FoxO signaling pathway	3	15	4.20E-02	Ccnd1, Egfr, Sirt1

## 4 Discussion

The successful application of antimicrobials has significantly increased the production and use of antimicrobials (Tiseo et al., 2020; Tian et al., 2021). Some intensively used antimicrobials interrupt mitochondrial proteostasis and physiology in animals (Wang et al., 2015) and also impact plant chloroplasts in terrestrial and aquatic plants (Carvalho et al., 2014; Minden et al., 2018); thus, they affect the ecosystems and biogeochemical processes (Zhou et al., 2021). FLO is widely used in veterinary clinics and aquaculture. It plays important roles in the prevention and control of bacterial diseases and effectively ensures the benefits of animal breeding. However, the application of FLO has increased interest in its toxic and side effects including hematopoietic toxicity, immunotoxicity, genetic toxicity, and embryonic toxicity in animals (Guan et al., 2011; Hu et al., 2014; Ren et al., 2017; Hu et al., 2020). Moreover, terrestrial and aquatic plants could take FLO up from the polluted environment and display marked mitonuclear protein imbalances and toxic effects (Zhang et al., 2020; Sharma et al., 2021). Previous studies indicate that FLO contaminants in the environment can enter the human body through the food chain and water, which leads to a disturbance of the microbiome in pregnant women and childhood obesity (Wang et al., 2017; Zhou et al., 2021). However, in the process of veterinary clinical application of FLO, we found that FLO exposure repressed chicken embryonic development and induced early embryonic death (Hu et al., 2020). The risk of embryonic toxicity caused by FLO cannot be ignored and clarifying the mechanism of embryonic toxicity caused by FLO is important to the research and development of new drugs and the scientific and rational application of the drug. In the present study, we find that FLO inhibits the proliferation, pluripotency, and cardiac differentiation of P19SCs, and DEGs and related pathways involved in FLO-induced toxic effects of P19SCs are identified.

We establish a FLO exposure model using P19SCs to evaluate the effect of FLO on the pluripotency, proliferation, and differentiation of ESCs. We find that FLO inhibits the proliferation of P19SCs (Figure 1A). P19SCs, which are the malignant counterparts of ESCs, are considered a good model to use to study stem cell biology (Vega-Naredo et al., 2014). ECSs can differentiate into specific cell types during embryonic development and postnatal growth (Lisowski et al., 2018). Studies prove the involvement of mitochondria in regulating stem cell behaviors, including self-renewal, proliferation, and differentiation (Chakrabarty and Chandel 2021). Mitochondrial dysfunction caused by chemicals might repress the full pluripotency and embryonic developmental potential of pluripotent stem cells (Liu et al., 2020; Paccola et al., 2021). Endosymbiotic theory and the similarity of ribosomal machinery between bacteria and mitochondria indicate that FLO can unsurprisingly affect mitochondrial protein synthesis, which is confirmed by our previous study (Hu et al., 2017). Thus, we speculated that FLO-induced embryonic toxicity may be related to the disrupted behaviors of stem cells caused by mitochondrial dysfunction, which needs to be further studied. Oct4, which is a transcription factor that is highly expressed in pluripotent cells, plays vital roles in regulating the induction and maintenance of pluripotent cells during embryonic development; the expression of Oct4 diminishes when the aforementioned cells lose pluripotency (Chen et al., 2008). Sox2 is a target gene of Oct4 and is also essential for pluripotent cell development. Loss of Oct4 or Sox2 is reported to cause loss of pluripotency of ESCs and embryonic lethality (Boyer et al., 2006; Lee et al., 2021). In this study, we find that FLO decreases the expression of Oct4 and Sox2 (Figure 1C), which implies that FLO represses the pluripotency of P19SCs. Moreover, given the essential roles of Oct4 and Sox2 in regulating cell cycle and proliferation of stem cells (Lee et al., 2016; Lee et al., 2021), we speculate that FLO-induced downregulation of pluripotent factors and proliferation inhibition in ESCs might be key reasons for poor embryo quality and early embryonic death post FLO treatment.

To further obtain the globally affected BP and signaling pathways in FLO-treated P19SCs, we performed RNA-Seq and bioinformatics analysis. Among the significantly enriched BP terms, 22 terms enriched by more than 50 DEGs were related to embryogenesis and development, including angiogenesis, embryonic organ development, regulation of vasculature development, and morphogenesis of organs (Figure 2C). According to our previous study, FLO restricts the development of heart, blood vessels, and organs of chick embryos (Hu et al., 2020), which is in accordance with the BP-enrichment results in FLO-treated P19SCs. Therefore, studying the mechanisms of FLO-induced embryonic toxicity using a P19SC model and the RNA-Seq results is reliable.

The KEGG pathway analysis suggests the involvement of the Wnt signaling pathway in mediating the toxic effects in FLO-treated P19SCs. The Wnt signaling cascade is an evolutionarily conserved regulator of development throughout the animal kingdom. Emerging evidence proves the important role of the Wnt signaling pathway in maintaining self-renewing and pluripotent capacities as well as in tissue morphogenesis and architecture (Nusse and Clevers 2017). β-catenin, which is the key switch in the canonical Wnt pathway, is stabilized and translocates to the nucleus to interact with and activate transcription factors for promoting Wnt target gene expression when the Wnt ligand binds to its receptors. In the absence of the Wnt ligand, cytoplasmic β-catenin is phosphorylated by the destruction complex, which results in subsequent ubiquitin-mediated proteolysis (Clevers and Nusse 2012). In this study, we found 33 DEGs enriched in the Wnt pathway and that the top 10 hub genes were key molecules of the canonical Wnt pathway (Figures 3C,D). Decreased levels of β-catenin in FLO-treated P19SCs and differentiated EBs were also found (Figures 4A,B), and chemical activation of the canonical Wnt pathway increased the protein level of β-catenin and proliferation of FLO-treated P19SCs (Figures 4D,E). Importantly, a reduction or loss of β-catenin impaired mesendodermal germ layer formation and neuronal differentiation (Lyashenko et al., 2011), repressed the formation of the retinal pigment epithelium and lens epithelium (Fujimura 2016), and led to EB formation deficiencies and embryonic lethality before gastrulation (Zhong et al., 2019). In this respect, our findings may help invite more detailed studies of the canonical Wnt pathway in FLO-induced stem cell and embryonic toxicity.

We also constructed a PPI network and performed module analysis with the identified DEGs. Identifying functional modules in PPI networks helps us to understand the cellular functional organization and the underlying cellular mechanisms (Haegel et al., 1995). GO analysis of the module genes reveals enrichment of upregulated DEGs mainly in protein ubiquitination (ontology: BP), SCF ubiquitin ligase complex (ontology: CC), and ubiquitin-protein transferase activity (ontology: MF). KEGG pathway analysis further reveals that the upregulated module genes were significantly enriched in ubiquitin-mediated proteolysis (Table 1), which indicates the involvement of ubiquitination in FLO-treated P19SCs. Ubiquitination is a posttranslational modification of proteins and participates in the selective degradation of most proteins to regulate transcription, signal transduction, cell-cycle control, and stem cell biology (Blount et al., 2020). Pluripotent cells commonly utilize ubiquitin-dependent proteolysis to regulate the levels of transcription factors related to pluripotency or specify a particular differentiation route. Actually, the Wnt pathway provides a remarkable case for illustrating the functions of ubiquitylation in regulating stem cell quiescence, self-renewal, and cell-fate determination through the ubiquitylation of β-catenin and others (Werner et al., 2017). Based on our results, we speculate that protein ubiquitination may be related to the FLO-induced lower level of β-catenin and eventually represses the pluripotency and proliferation of P19SCs. We also found that the downregulated module genes were principally enriched in DNA replication (ontology: BP), nucleus (ontology: CC), nucleotide binding (ontology: MF), and DNA replication and cell cycle pathways (Table 2). Accumulating evidence shows that DNA replication and cell cycle machinery directly regulate the pluripotency and proliferation of pluripotent cells (Liu et al., 2019). Therefore, our results further confirm the toxic effects and potential mechanisms of FLO on stem cell pluripotency and proliferation.

We identified the top 10 hub genes of the upregulated and downregulated DEGs by sorting the obtained score. We further performed KEGG pathway analysis via DAVID to verify the functions and mechanisms of screened hub genes. As a result, the top two signaling pathways annotated for the downregulated hub genes are cell cycle and DNA replication (Table 3). This finding is in accordance with the result of KEGG analysis using the identified module genes (Table 2). Importantly, more detailed studies are needed to further verify our findings.

## 5 Conclusion

We find that FLO repressed the pluripotency, proliferation, and DMSO-induced differentiation of P19SCs. Using various bioinformatics analysis, we identified significantly enriched BP terms, including angiogenesis and embryonic organ development post FLO treatment, which is consistent with our previous findings in a chick embryo model. Moreover, the enriched KEGG pathways, especially the canonical Wnt pathway, will help elucidate the mechanisms underlying FLO-induced proliferation and differentiation toxicity of P19SCs. Our research also analyzed modules and hub genes and found the involvement of ubiquitin-mediated proteolysis, DNA replication machinery, and cell cycle machinery in regulating the pluripotency and proliferation of FLO-treated P19SCs. However, further experiments are required to validate our findings *in vivo* and *in vitro* and elucidate the potential mechanisms underlying FLO-induced embryonic toxicity. In conclusion, our study presents the first application of RNA sequencing analysis to reveal DEGs involved in the inhibition of proliferation and differentiation in embryonic stem cells by FLO. The hub genes and enriched pathways will open up new ideas for the study of FLO-induced embryonic toxicity and provide an experimental basis for the scientific and rational application of FLO.

## Data Availability

The data sets presented in this study can be found in online repositories. The names of the repository/repositories and accession number(s) can be found below: https://ngdc.cncb.ac.cn/gsa; CRA005030
