# Supplementary material for: Molecular Mechanisms Underlying the Inhibition of Proliferation and Differentiation by Florfenicol in P19 Stem Cells: Transcriptome Analysis
Source: Front Pharmacol. 2022 Mar 29;13:779664. doi: 10.3389/fphar.2022.779664 (PMC9002123; doi:10.3389/fphar.2022.779664)
Supplement: Supplementary file 1 [file DataSheet1.PDF]

Supplementary Table 1. Real-time PCR primer pairs used in this study.

Gene name	Forward (5'to 3')	Reverse (5'to 3')
Wnt3	CTCGCTGGCTACCCAATTTG	CTTCACACCTTCTGCTACGCT
Wnt8a	ACGGTGGAATTGTCCTGAGCAT	ATGATGGCAGCAGAGCGGATG
Myc	AAATCCTGTACCTCGTCCGATT	CCACAGACACCACATCAATTTC
Notch1	CCCTTGCTCTGCCTAACGC	GGAGTCCTGGCATCGTTGG
Rarb	ACAATGCTGGCTTCGGTCCTCT	TTCCTCAAGGTCCTGGCGGTCT
Pax6	TACCAGTGTCTACCAGCCAAT	TGCACGAGTATGAGGAGGTCT
Ccnd1	CGTATCTTACTTCAAGTGCGTG	ATGGTCTCCTTCATCTTAGAGG

Supplementary Table 2. Summary statistics of clean reads in the cellular transcriptomes of P19SCs.

Sample	Con1	Con2	Con3	FLO1	FLO2	FLO3
Raw reads	42846674	44203340	44048612	47830148	45779072	45237658
Clean reads (%)	97.20	96.56	96.96	97.95	97.37	96.97
Clean bases (Gb)	6.25	6.4	6.41	7.03	6.69	6.58
Error rate (%)	0.02	0.02	0.02	0.02	0.03	0.03
Q30 (%)	94.48	94.56	94.49	94.4	94.11	94.08
GC (%)	51.14	52.49	52.52	52.38	52.33	52.46
Mapped reads	39581727	40349343	40482665	44218445	42438186	41620472
Exon (%)	95.42	96.10	96.07	95.38	96.47	94.52
Intron (%)	1.84	1.48	1.55	2.25	1.48	3.09
Intergenic (%)	2.75	2.42	2.38	2.38	2.05	2.39

Supplementary Table 3. Identification of hub genes among the upregulated and downregulated DEGs using Cytoscape.

Category	Gene symbol	David Gene Name	Log2FC	padj
Upregulated genes	ITPKB	inositol 1,4,5-trisphosphate 3-kinase B	0.72	1.22E-04
	EGFR	epidermal growth factor receptor	1.009	2.70E-07
	SIRT1	sirtuin 1	0.658	2.55E-04
	NOTCH1	notch 1	1.99	2.54E-37
	LAMP1	lysosomal-associated membrane protein 1	0.746	9.50E-12
	PAX6	paired box 6	3.624	3.05E-06
	ERBB2	erb-b2 receptor tyrosine kinase 2	0.94	5.01E-11
	ZBTB16	zinc finger and BTB domain containing 16	1.041	1.53E-07
	CTSD	cathepsin D	0.85	7.83E-11
	FBXL3	F-box and leucine-rich repeat protein 3	0.642	1.22E-03
downregulated genes	MYC	myelocytomatosis oncogene	-1.183	2.03E-13
	UBC	ubiquitin C	-0.687	4.81E-06
	CDC6	cell division cycle 6	-1.609	8.13E-31
	PCNA	proliferating cell nuclear antigen	-1.062	2.13E-18
	EXO1	exonuclease 1	-0.679	6.61E-06
	CCND1	cyclin D1	-0.64	2.96E-04
	MCM4	minichromosome maintenance complex component 4	-0.923	1.83E-09
	MCM3	minichromosome maintenance complex component 3	-0.793	6.29E-12
	MCM7	minichromosome maintenance complex component 7	-1.05	4.82E-21
	RRM2	ribonucleotide reductase M2	-1.553	7.24E-31

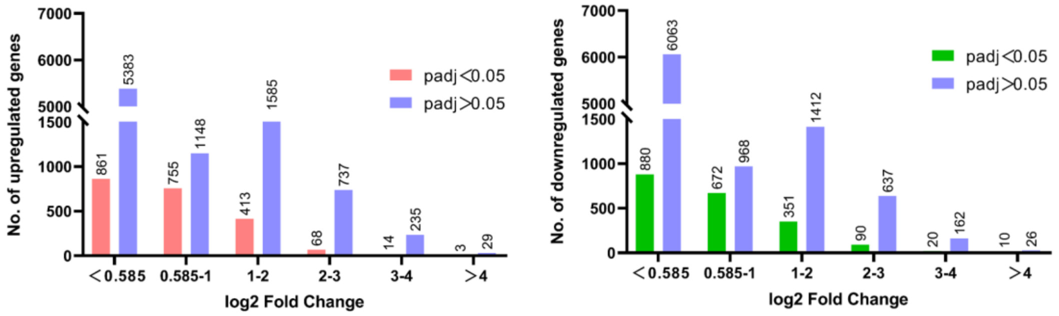

Supplementary Figure 1. Classification of the identified genes based on fold changes of expression level.

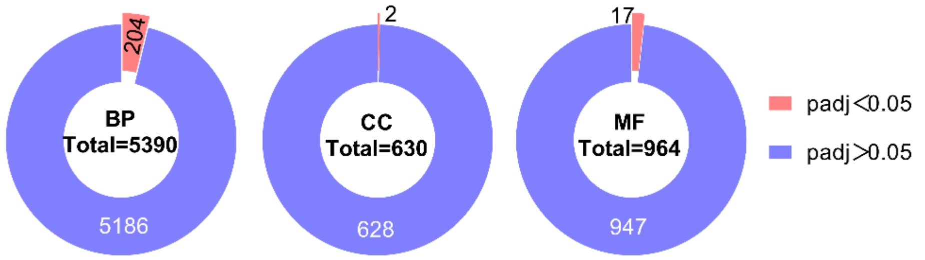

Supplementary Figure 2. Results of enriched biological process (BP), cell component (CC), and molecular function (MF). The number of significantly enriched terms are shown (padj <0.05).

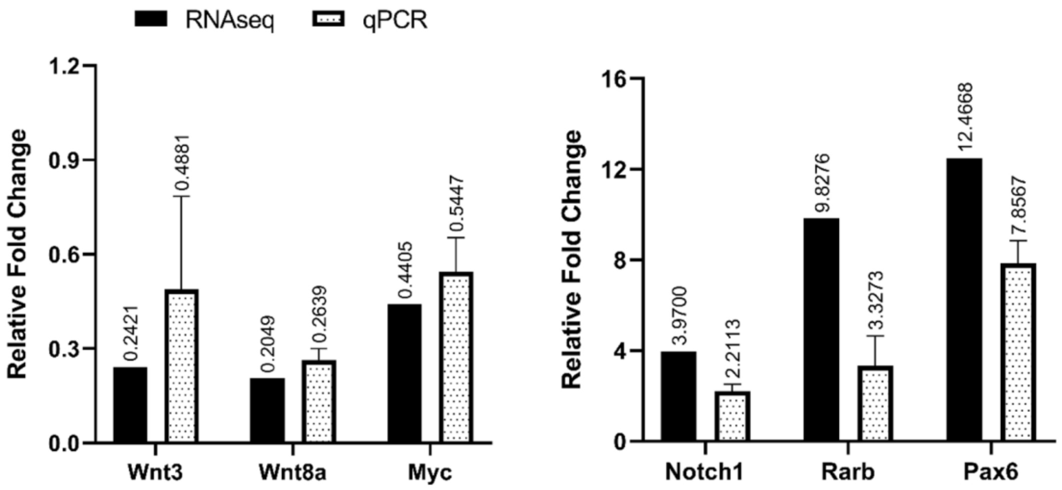

Supplementary Figure 3. Validation of the RNA-Seq results using qPCR. The expression levels of three downregulated DEGs (Wnt3, Wnt8a, and Myc) involved in the Wnt pathway and three other upregulated DEGs were detected using qPCR.

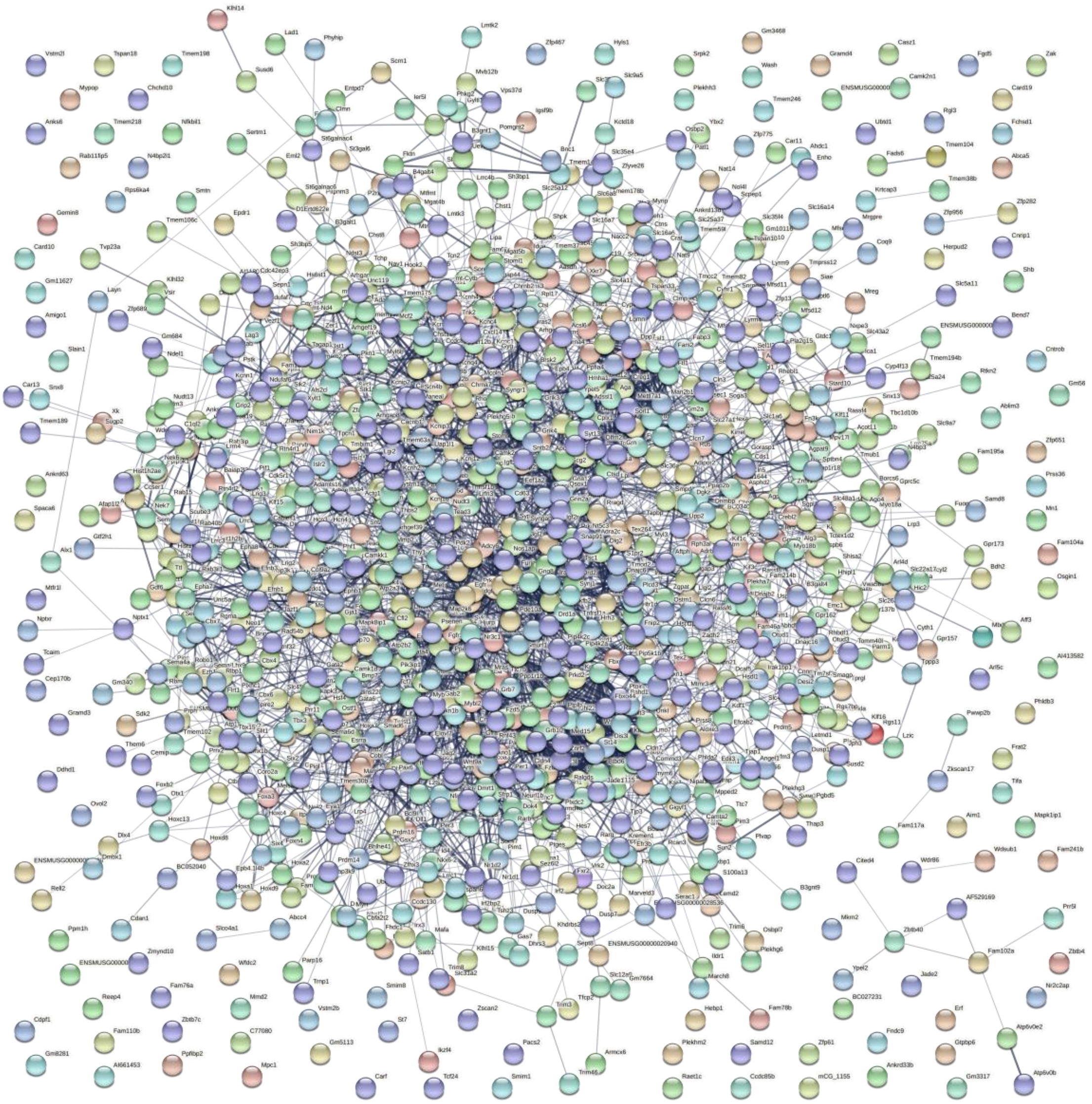

Supplementary Figure 4. PPI network analysis of the upregulated DEGs based on the String database.

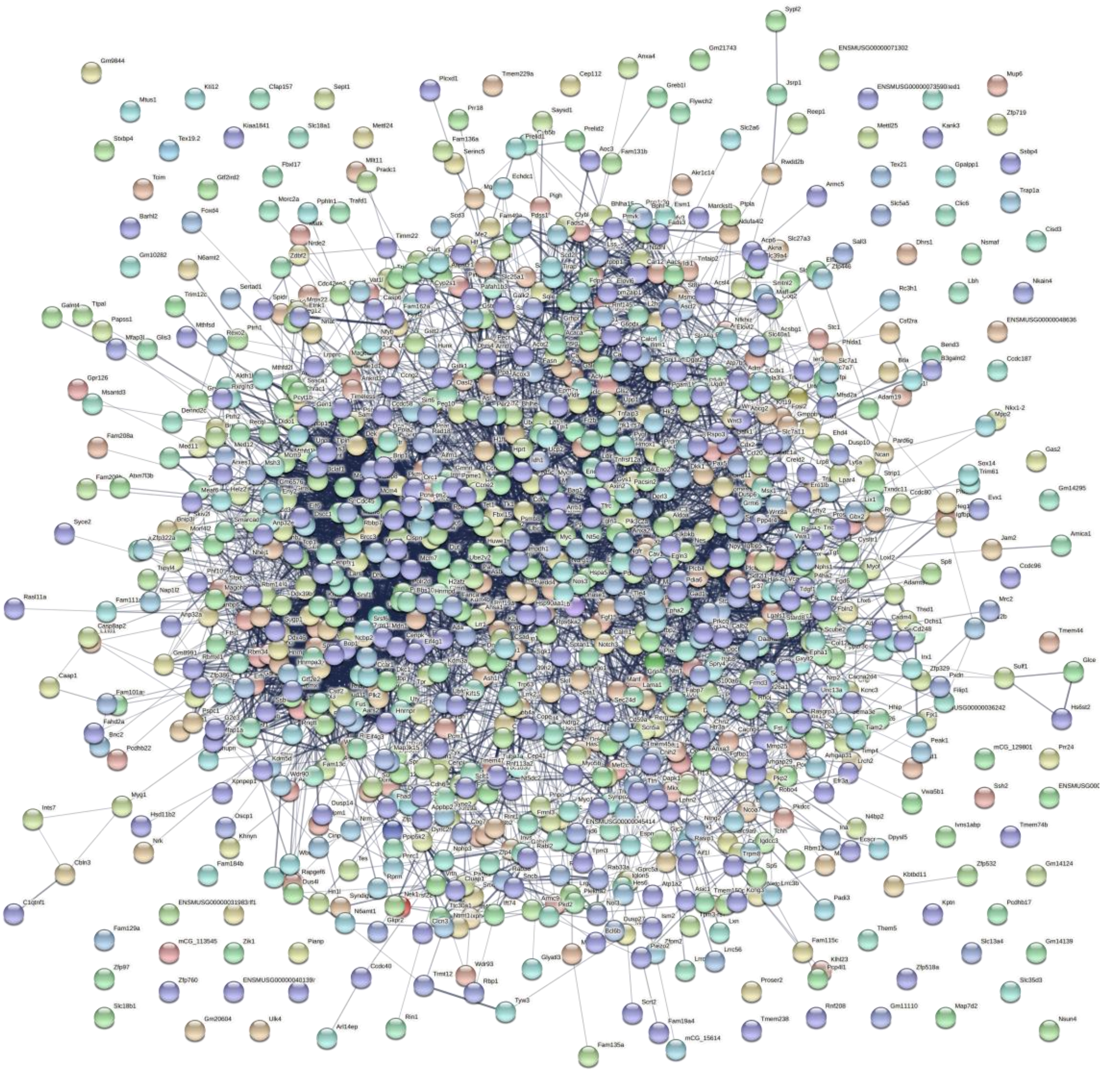

Supplementary Figure 5. PPI network analysis of the downregulated DEGs based on the String database.
